# Stakeholder perceptions of using “opt-out” for tobacco use treatment in a cancer care setting: a qualitative evaluation of patients, providers, and desk staff

**DOI:** 10.1186/s43058-023-00493-5

**Published:** 2023-09-20

**Authors:** Joshua W. Ohde, David O. Warner, Jason S. Egginton, Hildi J. Hagedorn

**Affiliations:** 1 Center for Digital Health, Mayo Clinic, Rochester, MN USA; 2 Department of Anesthesiology and Perioperative Medicine, Mayo Clinic, Rochester, MN USA; 3 Kern Center for the Science of Health Care Delivery, Mayo Clinic, Rochester, MN USA; 4 Center for Care Delivery & Outcomes Research, Minneapolis Veterans Affairs Health Care System, Minneapolis, MN USA; 5 Department of Psychiatry, University of Minnesota Medical School, Minneapolis, MN USA

**Keywords:** Opt-out, Tobacco, Cancer, Tobacco treatment

## Abstract

**Background:**

Continued tobacco use in cancer patients increases the risk of cancer treatment failure and decreases survival. However, currently, most cancer patients do not receive evidence-based tobacco treatment. A recently proposed “opt-out” approach would automatically refer all cancer patients who use tobacco to tobacco treatment, but its acceptability to cancer patients and providers is unknown. We aimed to understand stakeholder beliefs, concerns, and receptivity to using the “opt-out” approach for tobacco treatment referrals in a cancer care setting.

**Methods:**

Semi-structured interviews were conducted with oncology patients, providers, and desk staff. The sample size was determined when theoretical saturation was reached. Given the differences among participant roles, separate interview guides were developed. Transcripts were analyzed using standard coding techniques for qualitative data using the Consolidated Framework for Implementation Research (CFIR) codebook. Emergent codes were added to the codebook to account for themes not represented by a CFIR domain. Coded transcripts were then entered into the qualitative analysis software NVivo to generate code reports for CFIR domains and emergent codes for each stakeholder group. Data were presented by stakeholder group and subcategorized by CFIR domains and emergent codes when appropriate.

**Results:**

A total of 21 providers, 19 patients, and 6 desk staff were interviewed. Overall acceptance of the “opt out” approach was high among all groups. Providers overwhelmingly approved of the approach as it requires little effort from them to operate and saves clinical time. Desk staff supported the opt-out system and believed there are clinical benefits to patients receiving information about tobacco treatment. Many patients expressed support for using an opt-out approach as many smokers need assistance but may not directly ask for it. Patients also thought that providers emphasizing the benefits of stopping tobacco use to cancer treatment and survival would be an important factor motivating them to attend treatment.

**Conclusions:**

While providers appreciated that the system required little effort on their part, patients clearly indicated that promotion of tobacco cessation treatment by their provider would be vital to enhance willingness to engage with treatment. Future implementation efforts of opt-out systems will require implementation strategies that promote provider engagement with their patients around smoking cessation while continuing to limit burden on providers.

**Supplementary Information:**

The online version contains supplementary material available at 10.1186/s43058-023-00493-5.

Contributions to the literature

- Most cancer patients who use tobacco do not receive tobacco treatment. A proposed “opt-out” approach would automatically refer all to tobacco treatment, but its acceptability among patients and providers was unknown.
- We found that most patients and providers were generally supportive of this “opt-out” approach.
- Providers appreciated that the system placed no additional burden on their time; however, patients thought it was important that their provider highlight how quitting tobacco could help their cancer treatment.
- Future implementation of such opt-out models may require implementation strategies that promote provider engagement in the process while also keeping provider burden low.

## Background

Continued smoking after a cancer diagnosis increases the risk of cancer recurrence, secondary malignancies, treatment-related toxicities, treatment failure, and death [1–7]. Despite these consequences, multiple challenges remain for cancer patients to quit, including that they are not consistently referred to evidence-based tobacco treatment services [8]. Referral to treatment typically only occurs if patients express interest in quitting, making receipt of treatment entirely dependent on readiness to quit. However, recent evidence shows that assessing readiness to quit is not required for successful tobacco use treatment, [9] opening new possibilities for treatment referral. A new approach initially proposed by Richter and Ellerbeck involves using an “opt-out” approach in healthcare settings to refer patients who use tobacco to tobacco treatment [10]. The opt-out approach offers evidence-based treatment to all tobacco users, regardless of their readiness to quit [10]. Early evidence suggests that the opt-out approach may enhance the uptake of tobacco treatment and increase quit rates even if short-term during cancer treatment [11, 12]. Patients recently diagnosed or currently under treatment for cancer are uniquely positioned to make lifestyle changes that may improve their chances of a positive outcome. Capitalizing on this opportunity may be an excellent method to achieving tobacco cessation. Even if patients do not choose to quit, a tobacco treatment appointment can provide important information about how continued smoking affects the progress of disease and treatment, information that can allow patients to make informed decisions.

The National Cancer Institute has instituted a program to increase the provision of evidence-based tobacco treatment to all patients with cancer, the Cancer Center Cessation Initiative [13, 14]. As a participant, the Mayo Clinic Comprehensive Cancer Center proposed to design and implement an “opt-out” system that refers all cancer patients who use tobacco to the Mayo Clinic Nicotine Dependence Center (NDC), a long-standing resource for tobacco treatment [15]. As a part of the design process, the team first enlisted bioethicists to perform an analysis of the ethics of the opt-out approach, concluding that it met acceptable ethical standards [16]. Formative work was then undertaken to support the design of a system using the electric health record (EHR, Epic©) to refer all cancer patients who use tobacco to the NDC using the opt-out approach [15]. This work began with analysis of workflow in the cancer center outpatient practice, which involved three types of individuals: the desk staff, who bring the patients from the waiting area to the room and obtain or confirm basic demographic and health information, oncology providers (physicians or advanced practice registered nurses) who conduct the exam and document the clinical visit, and the cancer patients.

Principles of implementation science recognize that stakeholder feedback is important to improve the design and implementation of clinical interventions. The present study was conducted to assess stakeholder beliefs, concerns, and receptivity to using an opt-out approach for tobacco treatment referrals in a cancer setting, involving desk staff, providers, and patients as the primary stakeholders. The results of these interviews could not only provide information valuable to the design of this particular referral system, but also more generalizable insights that could inform the design and implementation of “opt-out” tobacco treatment in other healthcare settings.

## Methods

### Participants and recruitment

This study was deemed exempt by the Mayo Clinic Institutional Review Board. All interview participants, who were patients or employees at the Mayo Clinic Comprehensive Cancer Center, provided oral consent.

Oncology patients were identified by reviewing the electronic health records of those who were seen by a provider in Medical Oncology within the prior year. Other patient inclusion criteria included (1) age 18 to 89 years old; (2) identified as a current smoker (smoking every or most days); (3) diagnosed with cancer, and (4) English speaking. Exclusion criteria included major barriers to providing informed consent (e.g., dementia) or receiving end-of-life care (e.g., hospice or clinical prognosis of survival ≤ 1 year) unless such prognosis was identified after the interview began. Using a convenience sampling approach, eligible patients were recruited by mailed invitations. Oncology providers and desk staff were identified using a list of division staff and recruited by e-mail. Provider participants included physician oncologists and advance practice registered nurses with specialized training in oncology. Interviews with providers and desk staff were conducted during the pilot implementation of the “opt-out” referral system described elsewhere [15]. In this system, the desk staff identifies current tobacco users and refers these patients to the NDC without the need for provider involvement. After their clinical visit with the providers, desk staff again interact with the patient during “check-out” to schedule the NDC appointment.

### Procedures

Interviews were conducted in-person or telephone by two study members (JO, JE). Given the differences in roles of each stakeholder group, separate interview guides were developed (Additional Files 1, 2, and 3). The patient interview guide focused on understanding patient opinions and concerns regarding tobacco cessation during cancer treatment and the use of an opt-out approach in such setting. The desk staff guide focused on their attitude towards tobacco use treatment among cancer patients, their role in the opt-out process, responses they received from patients, and recommendations for improvement. The provider interview guide aimed to determine provider’s attitudes towards tobacco use treatment among cancer patients, potential problems or barriers to tobacco treatment, opinion on the opt-out approach, and importance of their patients attending tobacco treatment services.

Patient demographics including age, sex, state of residence, and cancer site were collected at the time of the interview. Demographic information for providers and desk staff included years in practice, sex, clinical practice location, and tumor site specialty. The sample size was determined when theoretical saturation was reached. Saturation of concepts occurs when representativeness and consistency of topics are achieved, meaning additional interviews are no longer contributing new information. Interviews were audio recorded and transcribed.

### Data analysis

Transcripts were reviewed and coded by the qualitative analyst team members (JO, HH). Transcripts were analyzed using standard coding techniques for qualitative data using the Consolidated Framework for Implementation Research (CFIR) codebook which includes five domains: Innovation Characteristics, Outer Setting, Inner Setting, Characteristics of Individuals, and Process [17, 18]. The coding strategy permitted for coding text to multiple CFIR domains if appropriate. Emergent codes were added to the codebook to account for themes not represented by a CFIR domain. Emergent codes included “Ethics,” “Timing,” “Provider Perceptions,” and “Suggestions.” These codes are described in Additional File 4.

As CFIR domains were designed to assess the perceptions of organizational members regarding implementation efforts, they are generally not applicable to the patient experience. Therefore, emergent codes were separately developed for patient interviews using an inductive thematic analysis approach to analyze and code interviews (Additional File 5) [17, 19]. The analyst team (JO, HH) read each transcript separately and identified initial themes, developed thematic codes, and coded directly on printed transcripts.

At least 50% of transcripts were coded separately by the analysts and then discussed jointly to develop a consensus on CFIR domains and patient codes. The remaining interviews were coded by JO individually. Coded transcripts were then entered into the qualitative analysis software NVivo to electronically label and organize the codes. NVivo was used to generate code reports that consolidated all quotes of a specific code for each stakeholder group. To avoid overrepresentation of individual opinions, only code reports that contained text from a minimum of four respondents from a participant group were analyzed. The primary qualitative analyst (JO) then reviewed each code report to generate preliminary themes and met with the secondary analyst (HH) to review, discuss, and reach a consensus on themes within each code. Once themes were identified, the analysts returned to the code reports to identify exemplar quotes that best represented each theme. Data is presented by stakeholder group and subcategorized by CFIR domains and emergent codes when appropriate.

## Results

### Patients

Invitations were mailed to 204 patients, of which 32 (16%) provided a response. An additional 59 (29%) patients were reached by follow-up phone calls. Theoretical saturation was obtained after interviewing 19 patients (Table 1). Of these, 63% were female and had a mean age of 62 (range 38 to 82 years). All but one participant was interviewed by phone.

**Table 1:** The demographics of patient participants, including sex, age, and primary cancer site

Participant ID (patients)	Sex (M/F)	Age (years)	Cancer site
P1	F	65	Skin
P2	F	74	Colon
P3	F	77	Lung
P4	M	59	Brain
P5	F	58	Kidney
P6	M	55	Brain
P7	F	54	Gallbladder
P8	M	64	Bladder
P9	F	72	Lung
P10	F	72	Sarcoma
P11	F	38	Pancreas
P12	F	67	Neuro
P13	F	70	Breast
P14	M	57	Liver
P15	M	52	Prostate
P16	M	38	Sarcoma
P17	M	55	Kidney
P18	F	82	Lung
P19	F	NA	Breast

*NA* data not available

#### Perceptions of smoking impact on cancer treatment

Many patients recognized that tobacco use increases cancer risk and believed their smoking caused their cancer. However, many were unsure how continued smoking might affect the course of their disease, making general comments that smoking “can’t help” or “it’s gonna hurt everything”, but not knowing information specific to their cancer about how continued smoking might affect treatment, morbidity, or mortality. Other motivators to quit or seek tobacco treatment included (1) requiring cancer surgery, with concerns about how continued smoking could affect postoperative healing, (2) prolonging life, whether or not a complete cancer cure was possible, (3) saving money by not buying tobacco products, and (4) improving long-standing respiratory symptoms such as cough or wheezing. Among reasons not to quit or seek treatment, most often mentioned was that if patients were told they had a short time to live, enduring the stress of trying to quit would not be worth it. In these circumstances, it seemed irrelevant to quit, especially if they still enjoy smoking. For example, a few respondents said that they already have cancer, or their cancer has metastasized, so “who cares?” As an example:

I mean I’ve already got brain cancer, I know the end’s coming, so the 5 cigarettes I smoke a day, how much is that shortening my life span? So, that’s the way I feel. That’s how I look at it, because if I completely quit smoking, I’m still gonna die of the brain cancer. It just makes me feel better through the day. (Patient 6)

Even for those with a better prognosis, some stated that continuing to smoke is not going to significantly shorten their life, or that quitting will not change their outcomes. Some patients also stated that smoking was an important tool to help them cope with the stress of having cancer and undergoing cancer treatment.

#### Provider communication about tobacco use and treatment

Patient reports about communication with providers about tobacco use or treatment were mixed. Only a few patients said their provider thoroughly discussed how continued smoking could impact their cancer care. Indeed, many patients reported that their provider said nothing about their smoking. When asked what would convince them to attend a NDC appointment, patients commonly stated that discussing smoking cessation in relation to their cancer was more important than discussing it in the context of general health, suggesting the importance of communicating a specific, time-relevant reason to quit smoking. As an overarching theme, patients felt that clear advice to quit and encouragement to seek treatment by the oncology provider would be important motivators to attend a NDC appointment. As a representative quote:

Interviewer: Would you approach it differently if your oncologist told you that they thought it was affecting your treatment?

Participant: You’re damn right I would. This is my life I’m talking about. If he said, hey the little bit of smoking you’re doing, hey it’s taking years away from the treatment you’re getting, I can go cold turkey in no time. (Patient 15)

#### Reactions to the “opt-out” approach

Patient reactions to the opt-out approach were sought using either a hypothetical situation explained by the interviewer or an actual patient experience. Approximately half of the patients expressed support for the systematic application of the opt-out approach to all cancer patients, while the others had concerns. Concerns included placing blame or guilt on patients or influencing patient autonomy as they perceived being forced into the referral, as represented below:

I don’t think that you should be automatically referred. I think that a person would feel… may feel as though they’re being forced into something that they’re not quite ready to do. (Patient 11)

Important rationales stated by the supporters included the needs of smokers for support, the concept that patients attending the NDC are not required to quit but can learn about the impacts of smoking and available treatment resources when they are ready, and that NDC attendance could motivate quitting, even among those not initially motivated to quit. One respondent explained that tobacco treatment should be part of the cancer care program:

Well, if it’s part of the whole cancer care program, they might be more likely to accept some counseling. If it’s left to them voluntarily, it might not be as… they might not so readily volunteer to do it, so if Mayo made it part of the program, I suppose they could still refuse, but if they make it part of the program for at least a one-time consultation, and then they could decide from there whether or not they wanted more. (Patient 2)

#### Potential barriers to attending the NDC

##### Costs

The potential for out-of-pocket costs for treatment was a potential barrier to accessing treatment. Patients wanted to know, prior to the appointment, if their insurance covers the cost of the appointment. Competing financial factors included out-of-pocket costs from their cancer treatment, decreased income from the inability to work, and living on a fixed income, such as Social Security or disability.

##### Feeling overwhelmed

Participants reported experiencing increased emotional stress, treatment burden, and generally feeling overwhelmed with appointments. Patients completing multiple appointments over several days reported fatigue:

I’m so glad I live here and Mayo’s here, but you get tired of appointments. You get really tired of going. (Patient 13)

Although some mentioned that the most overwhelming period was immediately following diagnosis and considered the least likely time patients would be willing to visit the NDC, others felt that treatment should happen at the time of diagnosis as patients may be motivated to change and early quitting could benefit treatment.

##### Unwillingness to change

Several themes emerged among participants who stated an overall lack of interest in attending an NDC appointment. A common theme was that patients viewed the NDC appointment as synonymous to entering tobacco treatment with the primary goal of quitting. Participants alluded to the lack of willingness to change, or readiness to quit, as a barrier to treatment. Simply put, if patients did not want to quit, they were not going to an NDC appointment.

### Desk staff

Seventeen (17) desk staff were invited to participate, of which 9 (53%) responded. Of respondents, 6 (3 female) agreed to participate and were interviewed. All interviews were conducted in-person.

#### Innovation characteristics

Overall, respondents were supportive of the opt-out system and believed there are clinical benefits to receiving information about tobacco treatment from the NDC and quitting smoking. Desk staff expected that providers would discuss the NDC appointment and tobacco use with patients during their oncology consultation. However, every desk staff participant stated that, from their perception, providers were not having these conversations with patients who received an NDC referral and believed providers need to engage with patients. For example:

The other day I had a head and neck [cancer] patient that came out, we set up the appointment, and I said, so we set up the appointment, and the doctor’s like, what appointment? And I’m like, well, the Nicotine Dependence, and the doctor turned around to the patient and said, I didn’t know you smoke. I’m like, it’s a head and neck patient, and… I’m like, that was awkward. (Desk staff 4)

#### Inner setting

The role of the desk staff is altered by the opt-out system; asking about tobacco use was already part of ordinary practice but placing a referral without a provider signing the order was a new responsibility. Desk staff expressed concerns whether they had the proper training to place the referral, talk to patients about the referral, or answer patient questions, as current institutional policy prohibits them from giving advice to patients as they are not medically certified. As one example:

If they’ve got questions, we’re not equipped to answer the questions, so I mean, you start a conversation that you can’t finish. (Desk staff 6)

#### Ethics and timing

There was a general belief that patients at end-of-life or in hospice should not be automatically referred to tobacco treatment. Participants stated potential ethical issues to automatically referring a dying patient to a service that was not life-prolonging and may cause additional emotional distress. This concern was based on the concept of poor timing as quality of life should be the priority and tobacco treatment should not be automatically presented. It was even thought to be too late, as summarized below.

They’ve already got enough on their plate to be thinking about. If you know that they’re on that stage for end-of-the-line, going-to-hospice kind of thing, is the one time that I really don’t necessarily think that it’s probably the best time. It’s like talkin’ about a colonoscopy at that time. It just doesn’t make sense anymore. (Desk staff 6)

Even for patients not at the end-of-life, overwhelming patients was another concern. Patients recently diagnosed or undergoing treatment are burdened with multiple appointments over several days. Some desk staff believed adding another appointment during this time may increase treatment burden and should be delayed until patients are less overwhelmed. Interestingly many of the same desk staff also discussed perceived clinical benefits to patients who quit during treatment.

### Providers

Twenty-six (26) providers were invited to participate, of which 23 (89%) responded. Of these respondents, 21 (91%) were interviewed across 4 Mayo Clinic sites: Rochester, Minnesota (*n* = 16), Mayo Clinic Health System in Minnesota (*n* = 2), Arizona (*n* = 2) and Florida (*n* = 1). Seven were interviewed by phone and 14 in-person. Among participants, 13 (62%) were male with an average of 15 years in clinical practice.

#### Innovation characteristics

A consistent theme across providers was the practical advantage of the intervention itself — an “automatic” referral that did not require their direct involvement to function (e.g., they did not need to place or co-sign the order); they were too busy to “click” another button. Time was considered the most valuable resource among providers. Also, they appreciated that the system had little effect on practice workflow. For example:

So as long as we don’t have to make some extra clicks, everything is happening automatically, we have no problem from the provider’s point of view. (Provider 6)

The nice thing is the way this was implemented, it had very little impact on the practice. (Provider 21)

#### Outer setting

Some providers were concerned that patients are too busy with treatment appointments or overwhelmed with a new diagnosis, thus lacking the time, energy, or desire to visit the NDC. Some felt that the referral should happen a few weeks or months after a patient’s initial diagnosis to lessen appointment burden. In contrast, other providers identified this as an opportunity to help patients in a devastating position, as summarized below:

But, in general, I think that the only other barrier is just another- is from the patients. Sometimes, we give ‘em really devastating news, and they’re crying when they’re leaving, and they really don’t wanna go to any other appointments, but that might be an opportunity to kind of schedule in the future or something like that. (Provider 11)

The need to respect patients who smoke and accept their tobacco use as a part of their lifestyle was another concern. Some mentioned that patients with cancer are stressed and that smoking acts as a stress reliever, such that quitting may be difficult in this situation. Patients should not be penalized for lifestyle choices. An example of this is shown below.

So, I think just the lesson from that being that patients certainly started to smoke for a variety of reasons. And while we as health care professionals certainly see that as detrimental to their health, we need to recognize that, for many people, smoking has become part of their lifestyle. It may be a stress reliever or something that it’s not so easy, sometimes, for them to quit, particularly in stressful situations, like a diagnosis of cancer. (Provider 21)

Some expressed that tobacco treatment for some patients may be futile:

It’s always good to stop, but, unfortunately, not to sound fatalistic, but in some ways, for these patients, it’s too late. So then you get the argument, ‘Well I’m gonna- I have a stage 4 bladder cancer. Why am I gonna stop? That’s an enjoyment in my life’. I don’t have a comeback to that when they’re gonna be dead in a year and a half. (Provider 16)

#### Inner setting

Providers generally appreciated the importance of tobacco use, and tobacco treatment, for their patients, recognizing that tobacco use may interfere with optimal cancer treatment:

It’s very important for our patients- probably all patients. But specifically for our patients, the treatment is very hard, and there is known documented studies that show that continued use of tobacco makes the treatment work less. It becomes less effective, the chances of having recurrence is high, and the healing from the treatment is also delayed. (Provider 14)

However, a few providers considered tobacco treatment to be less important compared to other treatment responsibilities.

#### Characteristics of individuals

Another theme included the lack of training oncologists have in tobacco treatment, and thus referring patients to the ‘experts’ seemed like the best idea. Providers also do not have the time to deliver tobacco treatment. Using a holistic approach, treating patients with multiple healthcare teams would provide comprehensive care and utilize Mayo’s resources in an efficient manner. One example of this theme is below:

…it’s not necessarily the core of what we do. It’s the core of what you do, but it’s not the core of what we do. And so I think bringing in those complimentary things, and I don’t have a lot of concern about taking away my control over whether or not someone addresses tobacco cessation with my patient. I’m willing to let that go. Let the experts do their job. (Provider 11)

#### Ethics

Like some desk staff, providers viewed the NDC referral irrelevant for end-of-life patients. It was said that patients in this stage of care should not be included in an automatic referral system for tobacco use, and instead, the provider should place the order if deemed appropriate:

If it’s somebody with incurable cancer but where we have treatments, that’s somebody I would see as, ‘Well yes, they have a terminal disease, but they’re apt to live maybe even a few years with the cancer’. And there, in that setting, it becomes really important to address the tobacco. But the truly terminal patient where we’ve kind of run out of options, they’re too sick for treatment, our focus is really palliation of symptoms. (Provider 21)

There was a consistent belief that there are few ethical problems with this approach if patients have the option to cancel or not attend the appointment without repercussions. Some providers even stated it is unethical to *not* have patients see a tobacco treatment specialist or disclose effects of continuing to smoke during cancer treatment as providers are “ethically bound” to encourage patients to quit. However, it was considered unacceptable to require a patient to quit or make visiting the NDC a condition of receiving care for their cancer. The response below shows this when one respondent was asked directly about potential ethical concerns.

I don’t have any concerns about the ethics of it. I mean it would be unethical for me not to tell them that their smoking is gonna screw everything up. Ethics- the ethics of this situation forces me to mandate that they see a smoking cessation counselor. They can say no. I mean they still have free will. There’s no ethical issue, as far as I’m concerned. But I’m ethically obligated to tell them that we cannot fix them if they continue to smoke. (Provider 7)

## Discussion

This work provided a vital understanding of stakeholder beliefs and concerns about and receptivity to using opt-out for tobacco treatment in a cancer setting, an approach that considers treatment of tobacco use an integral and regular component of cancer care.

Overall acceptance was high among all groups. Providers overwhelmingly approved of the approach used for the BPA process as it requires little effort from them to operate and saves valuable clinical time. Providers also appreciated the use of the NDC to provide treatment given their limited training in tobacco treatment and time to deliver treatment. Desk staff supported the opt-out system and believed there are clinical benefits to patients receiving information about tobacco treatment from the NDC and quitting smoking. Approximately half of patients expressed support for using an opt-out approach as many smokers need assistance but may not directly ask for it. Similar qualitative studies report proactive referral (opt-in & opt-out) programs for pregnant smokers were accepted by healthcare staff and patients [20, 21].

One common misconception among patients was that attending an NDC appointment required a willingness to quit smoking. It was difficult for many participants to understand the primary purpose of the NDC visit was to become informed about how tobacco use can impact cancer outcomes, and the options for managing tobacco use. Even when told the referral was primarily about education, quitting or the expectation to quit was perceived as the immediate purpose of being referred. This is likely an effect of a healthcare system that continuously advises patients to quit tobacco. Opt-out does not require quitting but provides available resources to all patients, regardless of their readiness to quit. Additional patient education during the referral process to overcome this misconception may improve patient’s willingness to engage, for at least one appointment, with the NDC. Facilitators of engagement included recognition by clinical personnel, and many patients, of the benefits of cessation, and specifically informing patients that cessation may improve their cancer outcomes. Additionally, integration into clinical workflow and making discussions of tobacco treatment a part of routine care is valuable for impressing on patients the importance of the issue.

Concerns that quitting will provide limited benefit and may increase patient distress and treatment burden, especially in late-stage disease, were a perceived barrier by participants. Referring patients to tobacco treatment who are expected to die within 6 months or under the care of hospice was of particular concern among clinical participants. Moreover, patients tended to say tobacco treatment is irrelevant if they were told life-prolongation was not an option. Although there are potential benefits of quitting, even in the later stages of the disease, respecting a patient’s lifestyle choice and focusing on palliation may be of greater importance. As a patient progresses towards death, symptom burden increases, and overall functional performance decrease [22, 23]. Some patients may stop smoking without intervention as symptoms increase, making tobacco treatment irrelevant. Participants in this study voiced a valid concern related to this topic indicating that individual patient prognosis and preferences should be considered. In response, providers were educated on the accumulating evidence of benefit of tobacco treatment even at later disease stages but informed that patients at end-of-life should be respected by avoiding referrals when potentially inappropriate or clinically irrelevant.

Adequate training and preparation of desk staff for patient interactions was a possible concern as placing a referral without provider approval was a new role for the desk staff at Mayo Clinic. A similar study also found that reception staff involved in a proactive referral process believed the reception may not be a suitable setting for placing referrals or discussing smoking with patients [20]. To improve confidence and fidelity the study team provided enhanced desk staff educational sessions pre- and post-implementation. After attending the sessions, most desk staff were confident they could complete the requirements to place a referral to the NDC. This was important as their acceptance of this role reassured the implementation team as the BPA was disseminated to other departments.

Although the BPA and referral require no engagement by providers, patients, and desk staff mentioned, it would be beneficial for oncology providers to explain why quitting is important for cancer outcomes and to encourage patients to complete the NDC appointment. The concept that “if my provider thinks tobacco treatment is important for my cancer care I will be more likely to go” was common and identified a potential method to empower patients to complete a tobacco treatment consultation. Enhanced provider education was utilized in attempting to increase provider engagement. Additionally, a reminder system was adopted to alert providers that their patient uses tobacco. As suggested by several providers, when a patient was identified as using tobacco, the desk staff would leave a physical card in the exam room to alert the provider and facilitate a discussion about tobacco use.

This finding highlights one of the key benefits of stakeholder engagement during intervention development. The original BPA was designed to not require any additional work on the part of the oncology providers as their time limitations were seen as a major barrier to implementing the intervention. A similar study in an inpatient hospital setting using an EHR-based “opt-out” system also identified BPA fatigue, time constrains, and competing priorities as primary barriers to adoption among providers [24]. However, patients were clear that they would be more likely to engage with the NDC if their oncology provider spoke with them about the impact of their smoking on their specific treatment course and encouraged them to attend. It is clear that the intervention will not be optimally impactful without commitment of oncology providers to at least engage in this conversation. As stated above, this finding led to refinements to the intervention which were intended to promote provider engagement while still making it as easy and efficient as possible for the provider.

This study had some limitations. First, participation in the interviews was voluntary. Potential interviewees who did not participate may have differing opinions from those who did. This is especially relevant to the patient participants as those who are unwilling to discuss tobacco treatment options may have strong and consistently different opinions about the opt-out approach. Second, while several patients and providers raised the issue of timing of opt-out treatment relative to a patient’s cancer severity or life expectancy, patient interviews were not compared based on these disease variables. Future work may explore in more detail patient’s specific opinions about when in their disease course they feel discussion of tobacco treatment becomes irrelevant or perhaps even harmful. Third, all interviews took place within a large, integrated health care system with easy access to tobacco treatment services within the system. Findings may not generalize to smaller private practices without these convenient referral options. Lastly, there is potential for interview/analyst bias as the lead investigator participated in both interviewing and analysis. This risk was addressed and limited by including a second interviewer and a second analyst both of whom are qualitative experts but not tobacco cessation researchers.

## Conclusion

Results from this study provided valuable stakeholder input that aided in the refinement and dissemination of the opt-out referral process across multiple Mayo Clinic Cancer Center sites. These results may also inform the design and implementation of other referral systems being developed at various cancer centers, as well as other healthcare settings. Initial experience has shown that the referral system is well-accepted by patients and staff and can significantly increase the proportion of cancer patients who engage in tobacco treatment [12, 15]. Perhaps the most interesting finding is the conflict between the providers’ desire to have a system that functions independent of them, adding no additional burden to their workload, and the patients’ desire for their provider to promote engagement with tobacco cessation treatment. Future efforts to implement opt-out systems may need to include implementation strategies that promote provider engagement with the process while still limiting additional burden on them. One possible modification to address this would be to embed a member of the tobacco treatment team within the clinic to be available for warm hand-offs. This has shown to be effective in engaging patients with other mental health interventions, would decrease provider concerns about their own lack of knowledge and training in the area, and would make it easier for a reluctant patient to receive some basic information about what engagement with tobacco treatment would entail.

### Supplementary Information

**Additional file 1. **Interview Guide: Evaluation of a Presumed Consent Model for Tobacco Treatment among Mayo Clinic Cancer Center Patients.

**Additional file 2. **Interview Guide: Evaluation of a Presumed Consent Model for Tobacco Treatment among Mayo Clinic Cancer Center Providers (desk staff).

**Additional file 3. **Interview Guide: Evaluation of a Presumed Consent Model for Tobacco Treatment among Mayo Clinic Cancer Center Providers.

**Additional file 4. **Additional emergent codes for provider interviews developed by JO and HH after reading transcripts separately and identifying emergent thematic codes.

**Additional file 5. **Codebook for patient interviews were developed by JO and HH after reading transcripts separately and identifying emergent thematic codes.

**Additional file 6. **Standards for Reporting Qualitative Research (SRQR)*.

## Data Availability

The full transcripts of the interviews are not publicly available to minimize the risk of participant identification.

## Abbreviations

NDC: Mayo Clinic Nicotine Dependence Center
BPA: Best Practice Advisory
CFIR: Consolidated Framework for Implementation Research
