# Supplementary material for: Stakeholder perceptions of using “opt-out” for tobacco use treatment in a cancer care setting: a qualitative evaluation of patients, providers, and desk staff
Source: Implement Sci Commun. 2023 Sep 20;4:117. doi: 10.1186/s43058-023-00493-5 (PMC10510286; doi:10.1186/s43058-023-00493-5)
Supplement: Supplementary file 1 — Additional file 1. Interview Guide: Evaluation of a Presumed Consent Model for Tobacco Treatment among Mayo Clinic Cancer Center Patients. [file 43058_2023_493_MOESM1_ESM.docx]

**Additional File 1.**

**Interview Guide: Evaluation of a Presumed Consent Model for Tobacco Treatment among Mayo Clinic Cancer Center Patients**

Thank you for your willingness to participate in an interview today. We appreciate your time.

This interview typically takes about 30-60 minutes, but if you need to leave earlier, please let us know.

Warm up:

Can you tell me about your experience here at Mayo?

Can you explain any lifestyle changes you made since your diagnosis? (*Diet, exercise, tobacco usage, alcohol consumption*)

**(A) Knowledge of how tobacco use affects cancer treatment**

1. How do you think smoking could affect your cancer treatment?

2) Do you think it is important to quit smoking during cancer treatment? Why or why not?

**(B) Attitudes and beliefs about tobacco treatment during cancer treatment**

General

1) What are (were) your biggest fears or concerns about quitting while getting treated for your cancer?

- *Is this the right time to quit?*
- *Specific good and bad things about quitting at this time?*

Attitudes regarding the “opt-out” process

1. We are thinking of creating a system where all cancer patients who use tobacco would be scheduled to meet with a specialist to talk about their tobacco use and how it affects their cancer. This would be part of the routine care for all cancer patients who use tobacco. How would you feel about a system like this?

- *What kind of information would you want to receive prior to the appointment?*
- *What would make you more or less likely to talk with a specialist?*
- *Is this something you think Mayo Clinic should do as part of routine care?*

1. Would you be willing to talk with a specialist even if you are not ready to quit?

**(C) General Information**

1) What is your age?

2) What state do you live in?

3) What type of cancer were you diagnosed with?

4) What stage was given at time of diagnosis? (*If applicable*)

Is there anything else that you would like to add?

Thank you again for taking the time. If you have any additional questions about the study, please do not hesitate to reach out to us.
