# Supplementary material for: Stakeholder perceptions of using “opt-out” for tobacco use treatment in a cancer care setting: a qualitative evaluation of patients, providers, and desk staff
Source: Implement Sci Commun. 2023 Sep 20;4:117. doi: 10.1186/s43058-023-00493-5 (PMC10510286; doi:10.1186/s43058-023-00493-5)
Supplement: Supplementary file 2 — Additional file 2. Interview Guide: Evaluation of a Presumed Consent Model for Tobacco Treatment among Mayo Clinic Cancer Center Providers (desk staff). [file 43058_2023_493_MOESM2_ESM.docx]

**Additional File 2.**

**Interview Guide: Evaluation of a Presumed Consent Model for Tobacco Treatment among Mayo Clinic Cancer Center Providers (desk staff)**

Thank you for your willingness to participate in an interview today. We appreciate your time.

This interview typically takes about 15-30 minutes, but if you need to leave earlier, please let us know.

Warm up:

Which tumor groups do you typically support?

**(A) Demographics**

1) How many years have you been in this position?

2) Does anyone in your family use tobacco? (ASK at the END of the interview)

**(B) Attitudes and beliefs of tobacco use/treatment among cancer patients**

1) How important is it to address a patient’s tobacco use as a part of their cancer treatment? Why?

2) Do you think cancer patients should talk with a specialist about their tobacco use, even if they are not yet ready to make a quit attempt? Why?

3) What do you think about the system (*opt-out*) we developed for referring patients to the NDC?

*Prompt*

- *Any concerns?*

4) Do you believe it is your role to ask the patient and place an order to the NDC for a consultation? Why?

**(C) Responses since implementation: Patients and providers**

1) How are patients responding to this approach?

2) What is the biggest barrier to getting patients to schedule an appointment and keep it?

3) How have the providers responded since we implemented this?

**(D) Implementation and desk staff engagement**

1) Do you feel like we adequately engaged with you (*the desk staff*) prior to implementing this?

**(E) Recommendations**

1) What recommendations or advice do you have for us to improve this?

Is there anything else that you would like to add?

Thank you again for taking the time. If you have any additional questions about the study, please do not hesitate to reach out to us.
