# Supplementary material for: Stakeholder perceptions of using “opt-out” for tobacco use treatment in a cancer care setting: a qualitative evaluation of patients, providers, and desk staff
Source: Implement Sci Commun. 2023 Sep 20;4:117. doi: 10.1186/s43058-023-00493-5 (PMC10510286; doi:10.1186/s43058-023-00493-5)
Supplement: Supplementary file 3 — Additional file 3. Interview Guide: Evaluation of a Presumed Consent Model for Tobacco Treatment among Mayo Clinic Cancer Center Providers. [file 43058_2023_493_MOESM3_ESM.docx]

**Additional File 3.**

**Interview Guide: Evaluation of a Presumed Consent Model for Tobacco Treatment among Mayo Clinic Cancer Center Providers**

Thank you for your willingness to participate in an interview today. We appreciate your time.

This interview typically takes about 30 minutes, but if you need to leave earlier, please let us know.

Warm up:

Can you tell me about your role here?

Can you tell me how patients get to tobacco cessation programs here? (Like the Nicotine Dependence Center)

**(A) Demographics**

1) How many years have you practiced in your specialty?

**(B) Attitudes and beliefs of tobacco use/treatment among cancer patients**

1) How important is it to address a patient’s tobacco use as a part of their cancer treatment? Why?

2) How do clinicians in your practice determine a patient’s readiness to quitting?

3) What are the potential problems of undergoing tobacco treatment during cancer treatment?

4) What is the biggest barrier in your practice to referring patients to a tobacco treatment service?

*(Lack of time, lack of experience, awkward conversations, fear that patients will become upset, burdensome for the patient, burdensome for you)*

5) Do you think cancer patients should talk with a specialist about their tobacco use, even if they are not yet ready to make a quit attempt?

6) What do you think about an opt-out approach (“automatic referral”) for referring your patients to tobacco treatment?

*Prompt*

- *Any concerns?*
- *Would you participate in this type of approach?*
- *Who should place the referral? (e.g., you, nurse, APN, consultant, desk operations staff)*

7) In your opinion, how do you think your patients would respond to this approach?

8) Do you believe it is the role of the provider to recommend the patient speaks with a specialist about their smoking?

*Prompt*

- *How strongly do you believe* ***you should*** *make the recommendation?*
- *How strongly* ***do you*** *make the recommendation?*

**(C) Awareness of the Nicotine Dependence Center**

1) How familiar are you with the Mayo Clinic Nicotine Dependence Center (NDC)?

*Prompt*

- *How familiar are you with the services they provide?*
- *Have you ever referred a patient to the NDC?*

**(D) Recommendations**

1) What recommendations or advice do you have for us to streamline the implementation process into your practice?

*Prompt*

- *What are your recommendations to increase fidelity?*

Is there anything else that you would like to add?

Thank you again for taking the time. If you have any additional questions about the study, please do not hesitate to reach out to us.
