# Supplementary material for: Stakeholder perceptions of using “opt-out” for tobacco use treatment in a cancer care setting: a qualitative evaluation of patients, providers, and desk staff
Source: Implement Sci Commun. 2023 Sep 20;4:117. doi: 10.1186/s43058-023-00493-5 (PMC10510286; doi:10.1186/s43058-023-00493-5)
Supplement: Supplementary file 4 — Additional file 4. Additional emergent codes for provider interviews developed by JO and HH after reading transcripts separately and identifying emergent thematic codes. [file 43058_2023_493_MOESM4_ESM.docx]

**Additional File 4.** Additional emergent codes for provider interviews developed by JO and HH after reading transcripts separately and identifying emergent thematic codes.

1. Timing – included references about the timing of the intervention in relation of the patient’s cancer care
2. Provider Perceptions – statements about what providers believed a patient’s perception would be of the intervention
3. Suggestions – recommendations made by participants for improvement or changes to the referral intervention
4. Ethical considerations – statements regarding the ethical considerations of the intervention or concepts the study team deemed possible topics of further ethical discussion
