# Supplementary material for: Stakeholder perceptions of using “opt-out” for tobacco use treatment in a cancer care setting: a qualitative evaluation of patients, providers, and desk staff
Source: Implement Sci Commun. 2023 Sep 20;4:117. doi: 10.1186/s43058-023-00493-5 (PMC10510286; doi:10.1186/s43058-023-00493-5)
Supplement: Supplementary file 5 — Additional file 5. Codebook for patient interviews were developed by JO and HH after reading transcripts separately and identifying emergent thematic codes. [file 43058_2023_493_MOESM5_ESM.docx]

**Additional File 5.** Codebook for patient interviews were developed by JO and HH after reading transcripts separately and identifying emergent thematic codes.

1. Cancer History/Status – patient’s description of their cancer diagnosis, prognosis, and treatment plan
   1. *Diagnosis*
   2. *Prognosis*
   3. *Treatment*
2. Tobacco history/status – patient’s tobacco use history, current tobacco use
3. Perceptions of smoking impact on cancer treatment – what patients perceive about smoking and its impact on their cancer treatment and outcomes, knowledge of tobacco use on cancer treatment
4. Smoking cessation treatment experiences – past experiences with smoking cessation and/or experiences with the NDC (if they ever went) – needs to contain specific treatment details
5. Provider communication regarding smoking and/or NDC appointment – level of communication from their oncology provider regarding their current tobacco use and/or the referral to the NDC. Can also contain statements regarding if the provider talked to the patient they may change their willingness to go do the NDC (example: If my doctor told me it was important I would go).
6. Smoking changes since diagnosis – changes made in tobacco use since cancer diagnosis/treatment
7. Motivations for change/no change – motivating factors to changing/not changing (quit/not quit) current tobacco use, perceived benefits and disadvantages of changing/not changing
   1. *Benefits (potential benefits)*
   2. *Downsides (potential downsides)*
8. Opt-out reactions – opinions and/or experiences to the opt-out approach (referring everyone without consideration of patient’s readiness to quit)
   1. *Opinion of theoretical scenario*
   2. *Reaction/opinion to actual experience*
9. Recommendations for improvement – patient recommendations for improvement on the opt-out approach, referral mechanism, or tobacco cessation treatment in general
10. Communication style – preferred method of communication from providers regarding their tobacco use, quitting, or the NDC appointment. Can also contain statements of *what* patients want to know before going to the NDC.
11. Barriers to accessing the NDC
    1. *Costs –* concerns with cost of the appointment and/or medications
    2. *Distance and time –* distance from Mayo or time needed to attend the appointment
    3. *Overwhelming –* feeling of being overwhelmed with cancer diagnosis/treatment
    4. *Willingness, mindset, perceived lack of benefit* – simply not wanting to go, feeling that it doesn’t matter at this point in their care, not caring
12. Ethical considerations – patient perceived ethical concerns or issues that implementors should think about regarding potential ethical concerns
